# The potential of *Klebsiella* and *Escherichia-Shigella* and amino acids metabolism to monitor patients with postmenopausal osteoporosis in northwest China

**DOI:** 10.1186/s12866-023-02927-5

**Published:** 2023-07-26

**Authors:** Zhuang Liang, Yuqi Hao, Lei Yang, Puwei Yuan, Wulin Kang, Tingting Liang, Bing Gu, Bo Dong

**Affiliations:** 1 Department of Rehabilitation Hospital Pain Ward, Xi’an Jiaotong University Affiliated Honghui Hospital, Xi’an, Shaanxi, 710054 China; 2 Department of Internal Medicine, Ordos Traditional Chinese Medicine Hospital, Ordos, 017000 Inner Mongolia China; 3 Department of Clinical Laboratory Medicine, Guangdong Provincial People’s Hospital (Guangdong Academy of Medical Sciences), Southern Medical University, Guangzhou, 510000 Guangdong China

**Keywords:** Postmenopausal osteoporosis, Bone mineral density, Gut microbiota, Fecal metabolites, Biomarker

## Abstract

**Background:**

Intestinal flora has been proposed to mediate the occurrence of postmenopausal osteoporosis (PMO). However, the mechanism by which microbes and their metabolites interactively promote PMO remains unknown.

**Methods:**

This study aimed to investigate changes in the intestinal flora and associated metabolites, and their role in PMO. 16S rRNA gene sequencing and metabolomics were performed to obtain postmenopausal women with osteopenia (lower bone mass, LBM), postmenopausal women with osteoporosis (OST), and healthy women as the control group.

**Results:**

We identified taxa-specific and metabolite differences in the intestinal flora of the participants of this study. The pathogenic bacteria *Klebsiella* (0.59% and 0.71%, respectively) and *Escherichia-Shigella* (2.72% and 4.30%, respectively) were enriched in the LBM and OST groups (*p* < 0.05)*.* Some short-chain fatty acid (SCFAs) producing bacteria, *Lactobacillus*, *Akkermansia*, *Prevotella*, *Alistipes*, and *Butyricicoccus*, were reduced in patients with LBM and OST compared to the control. Moreover, fecal metabolomic analyses suggested that the metabolites of indole-3-acetic acid and 7-ketodeoxycholic acid were altered in the LBM and OST groups compared to the control (*p* < 0.05). Enrichment analysis suggested that valine, leucine, and isoleucine biosynthesis; aromatic amino acid biosynthesis; and phenylalanine metabolism were significantly associated with the identified microbiota biomarkers and OST. Moreover, metabolite marker signatures distinguished patients in the OST from those in the control group with an area under the curve (AUC) of 0.978 and 1.00 in the negative and positive ion modes, respectively. Finally, we also found that the fecal level of interleukin-10 (IL-10) in the OST group was significantly lower than that in the control group and LBM group (*p* < 0.05), while tumor necrosis factor-α (TNF-α) and interleukin-6 (IL-6) were significantly higher in the OST group than that in the control group (*p* < 0.05).

**Conclusions:**

This study provides robust evidence connecting the intestinal flora and fecal metabolomics with PMO. Integrated metabolite and microbiota analyses demonstrated that in addition to dysregulated bacteria, indole-3-acetic acid, 7-ketodeoxycholic acid, and other metabolites can be used for the distinguish of LBM and PMO.

**Supplementary Information:**

The online version contains supplementary material available at 10.1186/s12866-023-02927-5.

## Introduction

Postmenopausal osteoporosis (PMO) was reported that increases the risk of fractures in postmenopausal women [1]. The most common complication associated with PMO is fragility fracture, which often occurs in non-traumatic or mildly traumatic conditions of the hip, femur, or spine, leading to pain, deformity, dysfunction [2]. Approximately 10% of the global human and more than 30% of PMO over the age of 50 suffer from osteoporosis [3]. Moreover, the first-year and second-year mortality rates for hip fractures are 17% and 12–20%, respectively [4]. Therefore, osteoporosis is a significant global public health, medical, and economic burden; however, the awareness of osteoporosis is low. Meanwhile, more studies are needed to allow the development of preventive strategies for osteoporosis in China.

Osteoporosis is diagnosed by bone imaging examination, ultrasound, biopsy, and metabolism biochemical index measurements [5]. However, it is difficult to perform large-scale screening and monitoring of osteoporosis. Early diagnosis and interventions to prevent PMO progression can also greatly reduce future healthcare costs, as most economic costs associated with PMO are incurred in advanced stages [6]. To date, several factors, including environmental factors, diet, lifestyle, hygiene, antibiotics, and probiotics, have been reported to contribute to the improvement of PMO [7]. However, at present, the available methods for the early prediction of PMO are limited and use only a few clinical parameters that may not reflect the heterogeneity and complexity of the disease. Thus, more convenient and non-invasive alternatives are required.

Alterations in the gut microbiota can drive the development of osteoporosis by regulating the immune system [8]. Various types of gut microbiota-targeted treatments can prevent the development of osteopenia and improve osteoporosis outcomes in humans [9]. For example, Li et al. reported that *Lactobacillus rhamnosus* GG can attenuate bone inflammation, inhibit bone loss, and reduce gut epithelial permeability in mice [10]. *Bifidobacterium longum*, *Lactobacillus paracasei*, and a mixture of *Lactobacillus paracasei* and *Lactobacillus plantarum* can decrease femoral bone loss and increase bone mineral density in rats [11]. A few studies have examined the role of intestinal flora in the occurrence of PMO; however, existing datas are inconsistent. He et al. reported that *Klebsiella*, *Morganella*, *Escherichia/Shigella*, *Enterobacter*, *Citrobacter*, *Pseudomonas*, *Succinivibrio*, and *Desulfovibrio* were enriched in women with PMO in Xiamen, China [12]. Ling et al. have observed that *Actinobacillus*, *Blautia*, *Oscillospira*, *Bacteroides*, and *Phascolarctobacterium* were positively associated with PMO in Guangzhou, China [13]. Mrinmoy et al. have suggested that *Actinomycetes*, *Eggerthella*, *Clostridium X1Va*, and *Lactobacilli* were more abundant in patients with PMO in Ireland [14]. To sum up, because of the differences in eating habits and climate conditions in each region, there was significant differences in the composition and structure of gut microbiota from different regions. Therefore, more studies should be conducted on the gut microbiota of patients with PMO from different regions to explain regional differences.

In addition, microbiota-associated metabolic pathways realted to the pathogenesis of osteoporosis. These metabolic pathways include enrichment pathways of lipopolysaccharide biosynthesis [15]; membrane transport, metabolism of tyrosine and tryptophan, valine, leucine, and isoleucine [13]; and metabolism of N-acetylmannosamine, deoxyadenosine, and adenosine [16]. However, the specific microbes and metabolites, as well as the mechanisms by which they interactively promote PMO, are still unclear. Thus, it is necessary to evaluate the mechanistic implications of the intestinal flora and their metabolites in PMO.

In the present study, we integrated the gut metabolomic and intestinal flora profiles of patients with postmenopausal osteopenia and osteoporosis in Xianyang, China, and compared them with those of healthy women. This study provides a valuable resource for understanding postmenopausal osteopenia and osteoporosis-specific microbiota/microbiome features and interactions and offers new insights into understanding postmenopausal osteopenia and osteoporosis.

## Results

### Analysis of clinical characteristics

**Fig. 1:**
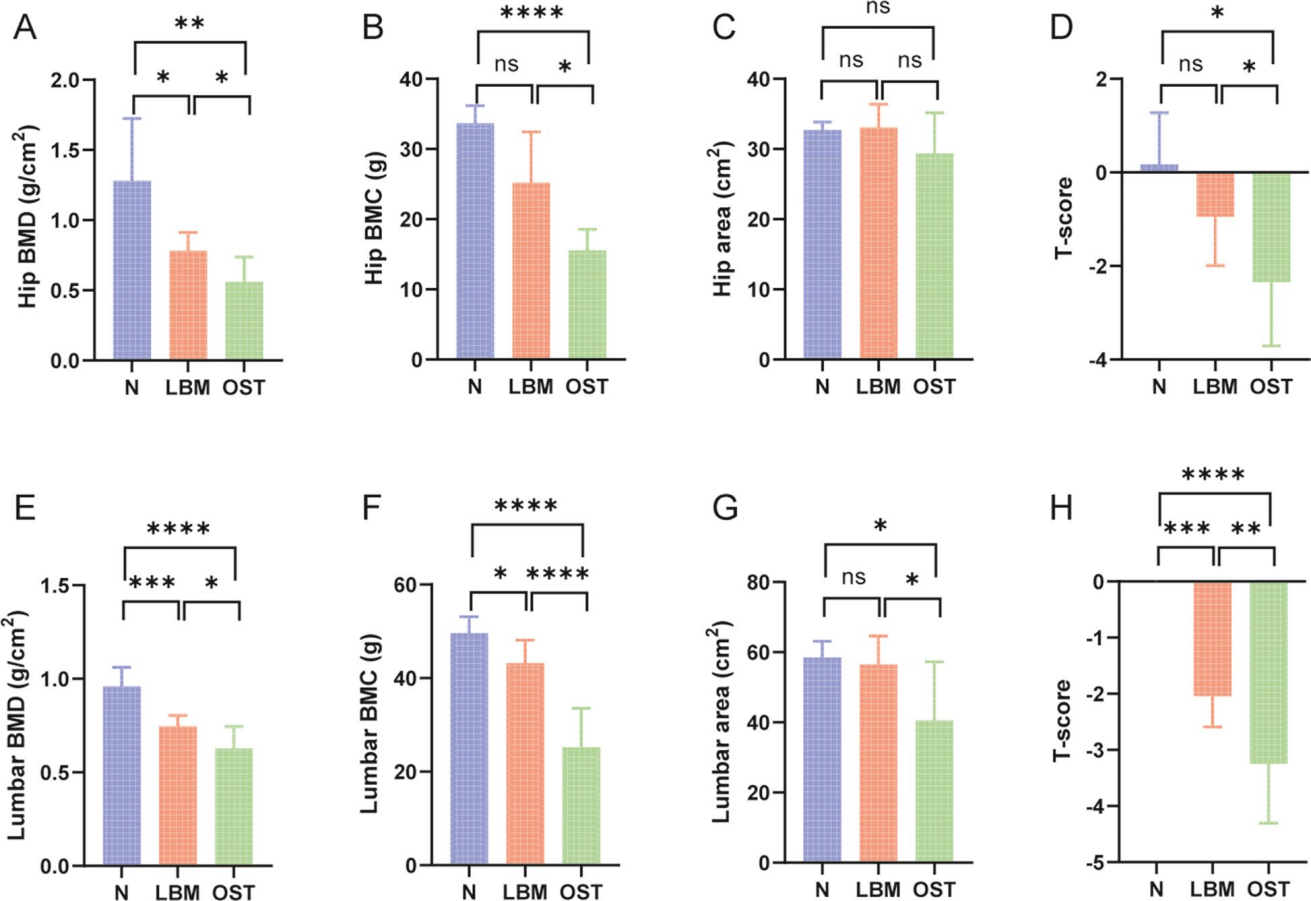
Clinical information of the participants. **A** Hip BMD. **B** Hip BMC. **C** Hip area. **D** Hip T-score. **E** Lumbar BMD. **F** Lumbar BMC. **G** Lumbar area. **H** Lumbar T-score. *p < 0.05, **p < 0.01, *** *p* < 0.001, **** *p* < 0.0001, ns represent no significant difference between each two groups

**Fig. 2:**
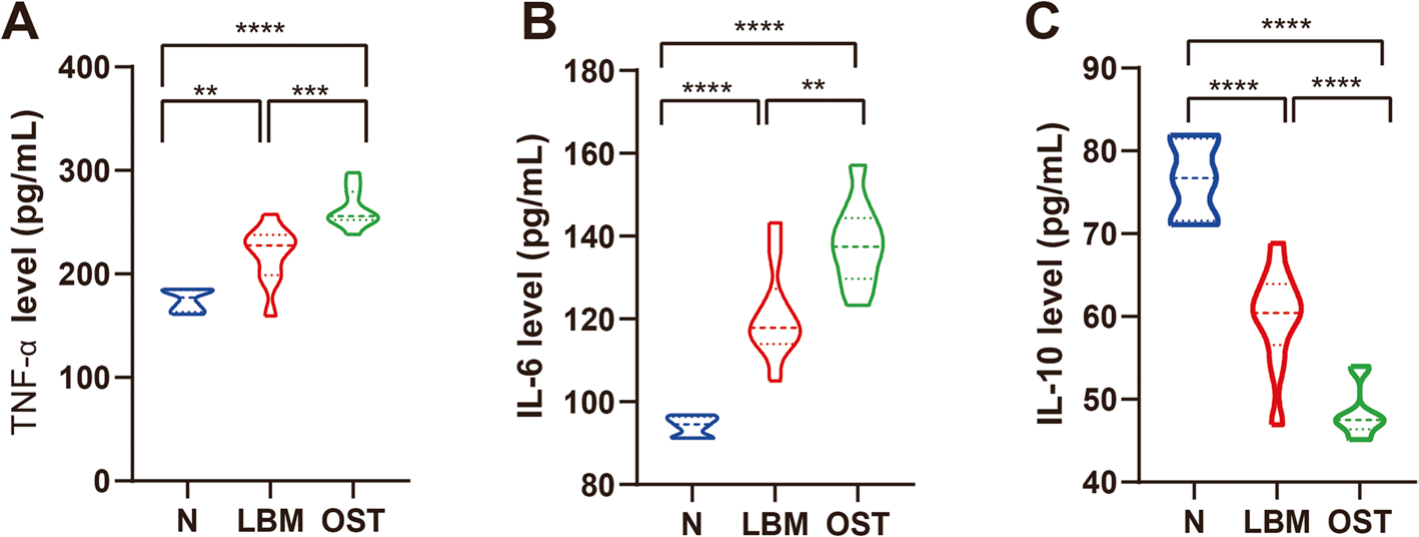
Changes in systemic inflammation status. Fecal levels of (**A**) TNF-α, (**B**) IL-6 and (**C**) IL-10. *p < 0.05, **p < 0.01, *** *p* < 0.001, **** *p* < 0.0001

Included 26 individuals were analyzed, the patients were evenly divided into healthy (control, *n* = 6), osteopenia (lower bone mass, LBM, *n* = 10), and osteoporosis (OST, *n* = 10) groups according to the bone mineral density (BMD) index. The hip BMD, hip bone marrow concentrate (BMC), and T-score were significantly lower in the OST group than in the control group (*p* < 0.05). Hip BMD in the LBM group was lower than that in the control group (*p* < 0.05). Hip BMD, BMC, and T-score were higher in the LBM group than in the OST group (*p* < 0.05); however, there was no significant difference in the hip area among these three groups (*p* > 0.05). Moreover, the lumbar BMD, BMC, area, and T-score were significantly lower in the OST and LBM groups than in the control group (*p* < 0.05). However, lumbar BMD, BMC, area, and T-score were higher in the LBM group than in the OST group (*p* < 0.05), and there was no significant difference in the lumbar area between the LBM and control groups (*p* > 0.05, Fig. 1). Furthermore, no significant differences were observed in age, weight, BMI, or menopausal period among the three groups (Table 1). In addition, the results in this study suggested that the level of the anti-inflammatory factor interleukin-10 (IL-10) was significantly lower in the OST and LBM group than in the control groups (Fig. 2C) (*p* < 0.05). Moreover, the level of the inflammatory factor tumor necrosis factor-α (TNF-α) and interleukin-6 (IL-6) were significantly higher in the OST group than in the LBM and control group (Fig. 2A, B) (*p* < 0.05).

**Table 1:** Clinical information of the participants

Participants, *n* = 26	Control (*n* = 6)	LBM (*n* = 10)	OST (*n* = 10)	*p*-value
Basic characteristics				
Age (years)	65 ± 2.9	66.9 ± 5.88	68.2 ± 5.9	0.5246
Weight (kg)	57.33 ± 5.75	62.4 ± 7.49	52.8 ± 13.93	0.1343
BMI (kg/m^2^)	21.37 ± 3.61	24.29 ± 2.54	22.09 ± 4.72	0.2660
Menopausal period (years)	50.33 ± 1.63	49.1 ± 4.65	49.4 ± 4.7	0.8481

### Characteristics of sample sequence and alpha and beta diversities of the gut microbiota

**Fig. 3:**
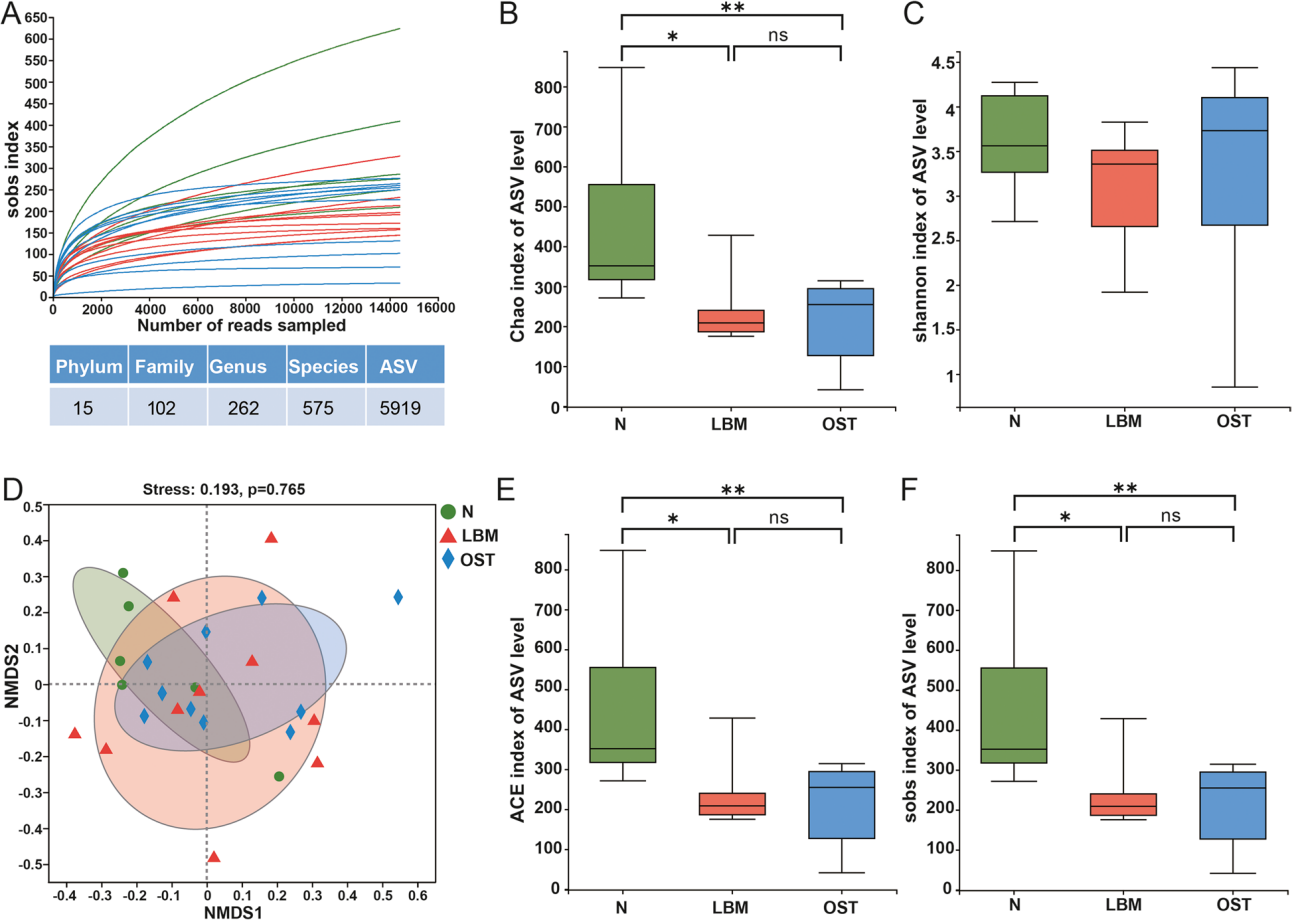
Alpha-diversity and similarity of bacterial community among the group of LBM, OST and N. **A** Sobs curve for each sample, and number of species in phylum, family, genus, species and ASV. **B** Chao index of ASV level. **C** Shannon index of ASV level. **D** Nonmetric multidimensional scaling analysis (NMDS) at the ASV level. **E** ACE index of ASV level. **F** Sobs index of ASV level. LBM: Lower bone mass, OST: osteoporosis, N: Normal group

A total of 5,236,251 high-quality reads were obtained from 26 samples, with a mean of 201,394.27 ± 27,004.13 sequences per specimen. The sobs index of curves for each sample were near saturation ((Fig. 3A), indicating that the sequencing data were sufficiently robust, with considerably few new undetected species. Moreover, the bacterial community richness indicated by the Chao1, ACE, and Sobs indexes were significantly lower in the OST group than in the control and LBM groups, and was significantly higher in the control group than in the LBM group, whereas the indexes were not significantly different between the LBM and OST groups (Fig. 3B, E, F). Similarly, the community diversity estimated using the Shannon index was not significantly different among the three groups (Fig. 3C). Additionally, nonmetric multidimensional scaling (NMDS) analysis for beta diversity had a differed of the bacterial, nevertheless, after anosim analysis, there were no significant difference among the control, LBM, and OST groups (*p* > 0.05)( Fig. 3D).

### Taxonomic composition of bacterial community

**Fig. 4:**
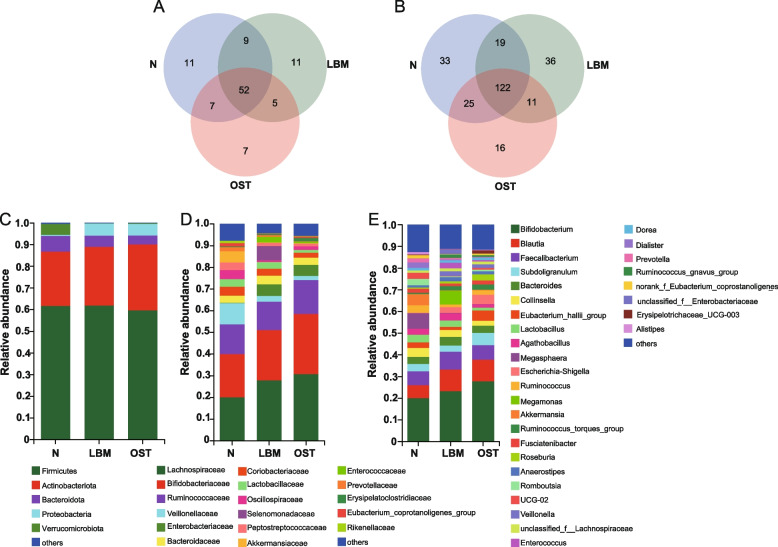
Composition of gut microbiota among the group of LBM, OST and N. **A** Venn diagrams of different groups in the bacterial biodiversity at the family level. **B** Venn diagrams of different groups in the bacterial biodiversity at the genus level. **C** Relative abundance of the bacteria community at the phylum level. **D** Relative abundance of the bacteria community at the family level. **E** Relative abundance of the bacteria community at the genus level. LBM: Lower bone mass, OST: osteoporosis, N: Normal group

We assessed the gut microbiome structure of the control, LBM, and OST groups using 16S rRNA gene sequencing, which generated a total of 15 phyla, 102 families, 262 genera, and 575 species across all samples. As shown in Fig. 4A, 79, 77, and 71 families were obtained from the control, LBM, and OST groups, respectively, among which 52 families were common in all samples. The OST samples had the lowest number of unique families (seven families). In addition, as shown in Fig. 4B, 199, 188, and 174 genera were detected in samples from the control, LBM, and OST groups, respectively, among which only 122 were common to all 26 samples. The control, LBM, and OST samples had 33, 36, and 16 unique genera, respectively.

At the phylum level (Fig. 4C), five major phyla, namely, Firmicutes, Actinobacteria, Bacteroidetes, Proteobacteria, and Verrucomicrobiota in all three sample groups. In most samples, Firmicutes and Actinobacteria were the two dominant phyla, with a total relative abundance accounting for 86.62%, 88.85%, and 90.00%, respectively. Moreover, Actinobacteria and Proteobacteria were enriched in the LBM (26.98% and 5.61%, respectively) and OST (30.39% and 5.40%, respectively) groups compared to the control group (25.01% and 0.004%, respectively), whereas Bacteroidetes and Verrucomicrobiota were depleted in the LBM (5.17% and 0.16%, respectively) and OST (4.13% and 0.30%, respectively) groups compared to the control group (7.36% and 5.15%, respectively).

At the family level (Fig. 4D), three major families. including *Lachnospiraceae*, *Bifidobacteriaceae*, and *Ruminococcaceae*, were identified in all samples. The relative abundances of *Lachnospiraceae*, *Bifidobacteriaceae*, and *Ruminococcaceae* in OST samples (30.75%, 27.70%, and 15.61%, respectively) were higher than those in LBM (27.80%, 23.18%, and 13.10%, respectively) and control samples (20.00%, 20.00%, and 13.72%, respectively). Moreover, the relative abundance of *Enterobacteriaceae* in the OST (5.02%) and LBM (5.39%) samples was higher than that in the control (0.21%) samples. However, control samples harbored other dominant families, including *Veillonellaceae*, *Coriobacteriaceae*, *Lactobacillaceae*, *Oscillospiraceae*, *Peptostreptococcaceae*, *Eubacteriumcoprotanoligenesgroup*, and *Akkermansiaceae*. Notably, other bacterial families, including *Selenomonadaceae*, *Enterococcaceae*, and *Erysipelatoclostridiaceae*, were also observed in LBM and OST samples but were not observed or were scarce in the control samples.

**Fig. 5:**
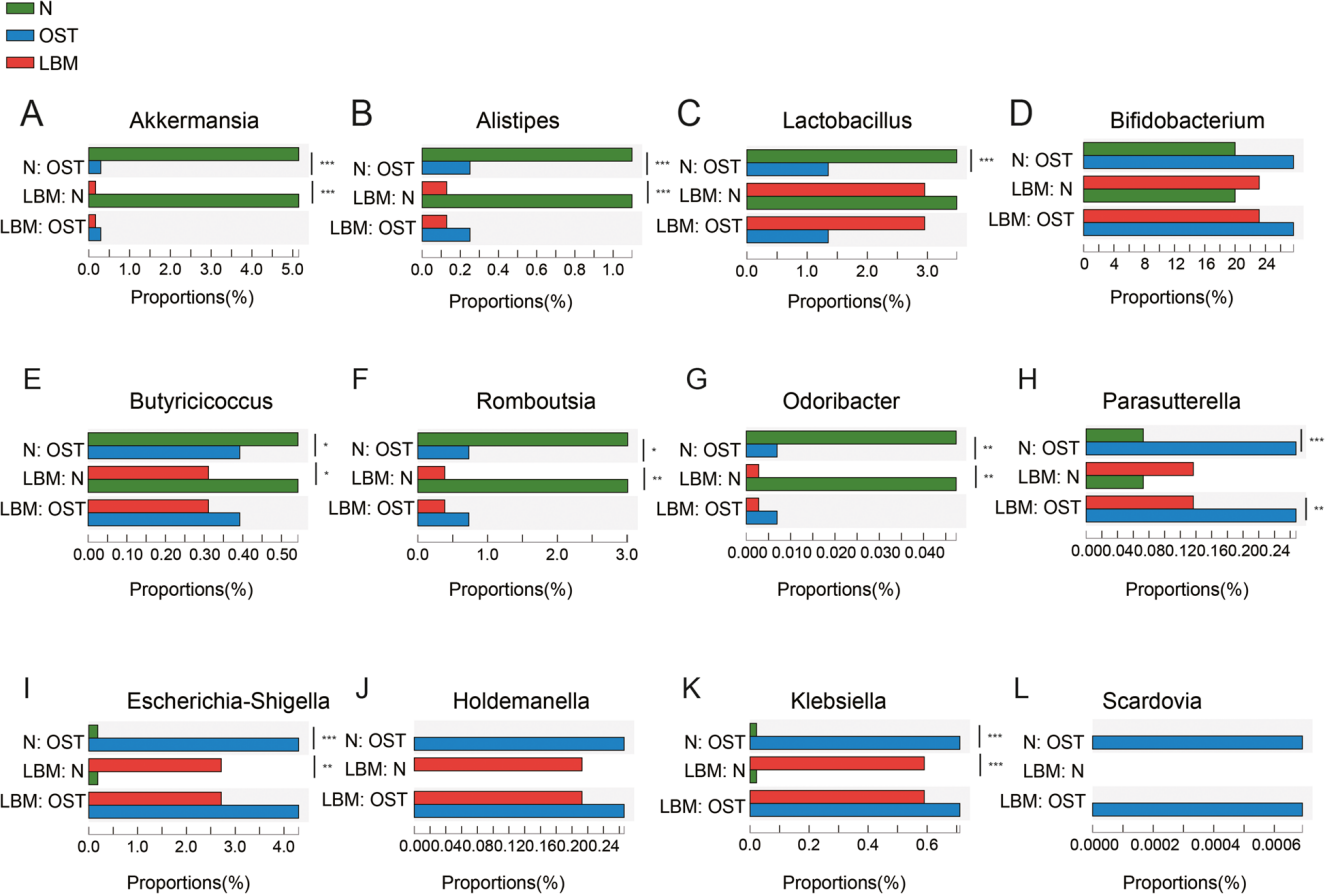
Difference analysis of microbial composition among the group of LBM, OST and N. **A***Akkermansia*. **B***Alistipes*. **C***Lactobacillus*. **D***Bifidobacterium*. **E***Butyricicoccus*. **F***Romboutsia*. **G***Odoribacter*. **H***Parasutterella*. **I***Escherichia-Shigella*. **J***Holdemanella*. **K***Klebsiella*. **L***Scardovia*. LBM: Lower bone mass, OST: osteoporosis, N: Normal group

At the genus level (Figs. 4E and 5), *Bifidobacterium*, *Blautia*, *Subdoligranulum*, *Ruminococcus_torques_group*, *Roseburia*, *Eubacterium hallii group*, and *Erysipelotrichaceae*_UCG-003 were the most abundant in the OST and LBM groups. The relative abundance of *Escherichia-Shigella* was higher in the LBM and OST groups (2.72% and 4.30%, respectively) but was not observed or was scarce in the control (*p* < 0.05). Several bacterial species, including *Lactobacillus*, *Megasphaera*, *Ruminococcus*, *Romboutsia*, *Akkermansia*, *Ruminococcaceae*_UCG-02, *Dialister*, *Prevotella*, and *Alistipes*, were enriched in the control group compared to the LBM and OST groups (*p* < 0.05). Moreover, *Megamonas*, *Anaerostipes*, and *Enterococcus* were abundant in the LBM group whereas *Erysipelotrichaceae*_UCG-003 and *Scardovia* were enriched in the OST group. Furthermore, the relative abundances of *Butyricicoccus* and *Odoribacter* were higher in the control group than in the LBM and OST groups whereas those of *Parasutterella*, *Holdemanella*, and *Klebsiella* were higher in the LBM and OST groups (*p* < 0.05).

### Fecal metabolite profiles

**Fig. 6:**
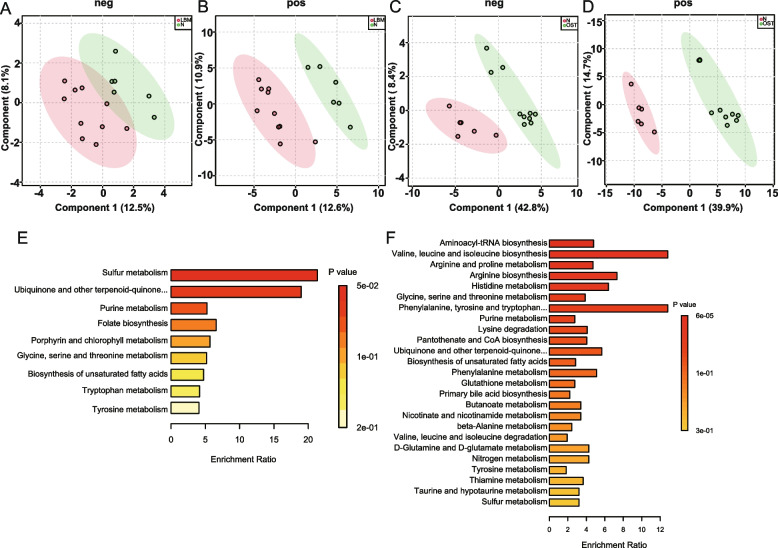
Metabolomic data profiles and pathway enrichment analysis. **A** Partial least squares-discriminant analysis (PLS-DA) for LBM and N groups in negetive model. **B** Partial least squares-discriminant analysis (PLS-DA) for LBM and N groups in positive model. **C** Partial least squares-discriminant analysis (PLS-DA) for OST and N groups in negetive model. **D** Partial least squares-discriminant analysis (PLS-DA) for OST and N groups in positive model. **E** Enrichment analysis altered metabolites between LBM and N. **F** Enrichment analysis altered metabolites between OST and N. LBM: Lower bone mass, OST: osteoporosis, N: Normal group

To assess whether the fecal metabolite profiles were associated with osteoporosis, we performed metabolic profiling of all stool samples. A total of 97 metabolites were quantified from stool samples using liquid chromatography-mass spectrometry (LC–MS). Partial least squares discriminant analysis (PLS-DA) (Fig. 6A-D) showed that there were differences in the gut metabolite profiles between the LBM and control group, OST and control group, after the fivefold cross validation, results shown that Q2 is all greater than 0.4, indicating the model is effective and a gut metabolite shift in the patients with osteoporosis.

The results revealed 15 significantly altered metabolites between the LBM and control groups; metformin, ornithine, stearic acid, amide C18, ursolic acid, 7-ketodeoxycholic acid, deanol, calcitriol, levalbuterol, hesperidin, and hypoxanthine were enriched in the LBM group, whereas dihydropteroate acid, diethyl phosphate, ofloxacin, and stearamide were depleted in the LBM group compared to the control group (Table 2). Moreover, 63 significantly altered metabolites were identified between the OST and control groups; metabolism of amino acids (L-proline, L-threonine, L-serine, L-phenylalanine, proline, and isoleucine) and organic acids (crotonic acid and stearic acid) was enriched in the OST group whereas that of (E)-ferulic acid, DL-tryptophan, L-( +)-leucine, L-tyrosine, L-theanine, creatine, and L-isoleucine was downregulated in the OST group compared to the control group (Table 3). Interestingly, the levels of 7-ketodeoxycholic acid (7-KDCA) and indole-3-acetic acid (IAA) were altered in the OST group compared to the control group, suggesting they have a potential impact on the development of osteoporosis.

**Table 2:** Important features identified by volcano plot in LBM & N

	Compounds	FC	log2(FC)	pval	trends
1	Dihydropteroic acid	0.41853	-1.2566	0.037848	down
2	Metformin	17.485	4.1281	0.0057087	up
3	Diethyl phosphate	0.41796	-1.2586	0.014181	down
4	Ornithine	3.2711	1.7098	0.017052	up
5	Stearic acid	8.4884	3.0855	0.018507	up
6	Ofloxacin	0.4266	-1.229	0.024241	down
7	Stearamide	0.48632	-1.04	0.02806	down
8	Amide C18	5.5551	2.4738	0.035269	up
9	Ursolic acid	5.1981	2.378	0.037459	up
10	7-ketodeoxycholic acid	5.668	2.5028	0.038545	up
11	Deanol	5.801	2.5363	0.041853	up
12	Calcitriol	5.051	2.3366	0.043178	up
13	Levalbuterol	4.6834	2.2276	0.046517	up
14	Hesperidin	4.7183	2.2383	0.048639	up
15	Hypoxanthine	5.2503	2.3924	0.048701	up

**Table 3:** Important features identified by volcano plot in N & OST

	Compounds	FC	log2(FC)	p value	trends
1	Phosphoarginine	12.551	3.6498	8.7424e-06	up
2	Xanthine	8.5894	3.1026	0.00010962	up
3	Aminolevulinic acid	12.474	3.6409	0.001194	up
4	o-Succinylbenzoate	0.30746	-1.7015	0.0019324	down
5	4-Nitrophenol	7.6822	2.9415	0.0025816	up
6	Dihydropteroic acid	4.4263	2.1461	0.0051274	up
7	2-Methoxyestrone 3-sulfate	3.2382	1.6952	0.0093508	up
8	2-(6’-methylthio)hexylmalic acid	0.46843	-1.0941	0.011958	down
9	Zalcitabine	0.41295	-1.276	0.013035	down
10	3,4-Dihydroxyphenylglycol O-sulfate	9.3213	3.2205	0.013586	up
11	Norepinephrine sulfate	0.22231	-2.1693	0.029922	down
12	fructosylglycine	2.7231	1.4453	0.031641	up
13	tetrathionic acid	2.1023	1.0719	0.033801	up
14	(E)-Ferulic acid	2.0952	1.0671	0.048625	up
15	L-Proline	0.001889	-9.0482	7.2329e-12	down
16	Irbesartan	0.00075929	-10.363	3.7902e-11	down
17	Phloionolic acid	0.0028703	-8.4446	1.3835e-10	down
18	Enalapril	0.0081559	-6.9379	8.6221e-10	down
19	L-Threonine	0.0060897	-7.3594	2.771e-09	down
20	DL-Serine	0.0042373	-7.8826	5.8641e-09	down
21	Amoxicillin	0.011054	-6.4993	4.104e-08	down
22	urobilinogen	19.09	4.2548	9.771e-08	up
23	Pipecolic acid	0.012253	-6.3508	2.6359e-07	down
24	N-Acetylhistamine	0.020567	-5.6035	8.5136e-07	down
25	Crotonic acid	0.030598	-5.0304	5.1701e-06	down
26	Aminolevulinic acid	7.5296	2.9126	8.6311e-06	up
27	Isoliquiritigenin	0.072636	-3.7832	9.9385e-06	down
28	Cinnamic acid	14.089	3.8165	1.3218e-05	up
29	DL-Tryptophan	16.092	4.0082	1.734e-05	up
30	(-)-codeine	7.5433	2.9152	1.7647e-05	up
31	Ofoxacin	17.909	4.1626	3.4116e-05	up
32	2,3,4,5-tetrahydrodipicolinic acid	39.965	5.3207	4.278e-05	up
33	D-Alanyl-D-alanine	4.6462	2.2161	6.0808e-05	up
34	Thymine	14.686	3.8764	6.0812e-05	up
35	g-Butyrobetaine	5.2098	2.3812	6.7153e-05	up
36	Daidzein	12.957	3.6957	6.8347e-05	up
37	Stearic acid	0.1786	-2.4852	7.4136e-05	down
38	L-Phenylalanine	0.1954	-2.3555	9.463e-05	down
39	Cholest-4-en-3-one	15.548	3.9586	0.00023261	up
40	L-( +)-Leucine	38.539	5.2683	0.00023661	up
41	L-Tyrosine	2.9931	1.5817	0.00023666	up
42	Styrene	0.047016	-4.4107	0.00030534	down
43	L-Theanine	56.025	5.808	0.00053619	up
44	Acetylcholine	2.6748	1.4194	0.00054292	up
45	cholic acid	5.7303	2.5186	0.00061697	up
46	glutaral	0.11453	-3.1262	0.0007078	down
47	Indole-3-acetic acid	2.6714	1.4176	0.00097212	up
48	3-(3,4-dihydroxypheny]) propanoic acid	2.6031	1.3803	0.0013811	up
49	Chrysin	161.83	7.3383	0.001399	up
50	Tiglic acid	2.6267	1.3932	0.0014194	up
51	Creatine	144.07	7.1706	0.0015611	up
52	L-Isoleucine	2.9603	1.5657	0.0016086	up
53	Deanol	0.30279	-1.7236	0.0016419	down
54	7-ketodeoxycholic acid	0.30624	-1.7073	0.0017631	down
55	Proline	0.028507	-5.1325	0.0018182	down
56	Amide C18	0.32731	-1.6113	0.0026659	down
57	Hypoxanthine	0.34182	-1.5487	0.0028131	down
58	Isoleucine	4.2212	2.0777	0.0028639	down
59	Ursolic acid	0.35954	-1.4758	0.0031063	down
60	Berberine	101.27	6.662	0.0034375	up
61	Diethyl phosphate	0.22342	-2.1622	0.0036987	down
62	Glycochenodeoxycholic acid	2.5592	1.3557	0.0039483	up
63	N(1)-acetylspermidine	0.087937	-3.5074	0.0041961	down

To further understanding the functions of these significantly changed metabolites, we conducted enrichment and pathway analyses (Fig. 6E–F). Several metabolite sets and pathways were enriched in the LBM group, including sulfur metabolism, purine metabolism, folate biosynthesis, porphyrin and chlorophyll metabolism, glycine, serine and threonine metabolism, tryptophan metabolism, and tyrosine metabolism, compared to the control group. The top four enriched sets and pathways in the control group compared with the OST group were aminoacyl-tRNA biosynthesis, valine, leucine, and isoleucine biosynthesis, arginine and proline metabolism, arginine biosynthesis, histidine metabolism, and tryptophan biosynthesis, indicating that they were significantly downregulated in the OST group. These results suggest that metabolic pathways but not individual metabolites are altered in osteoporosis.

### Difference of metabolites between the group of osteopenia and osteoporosis

**Fig. 7:**
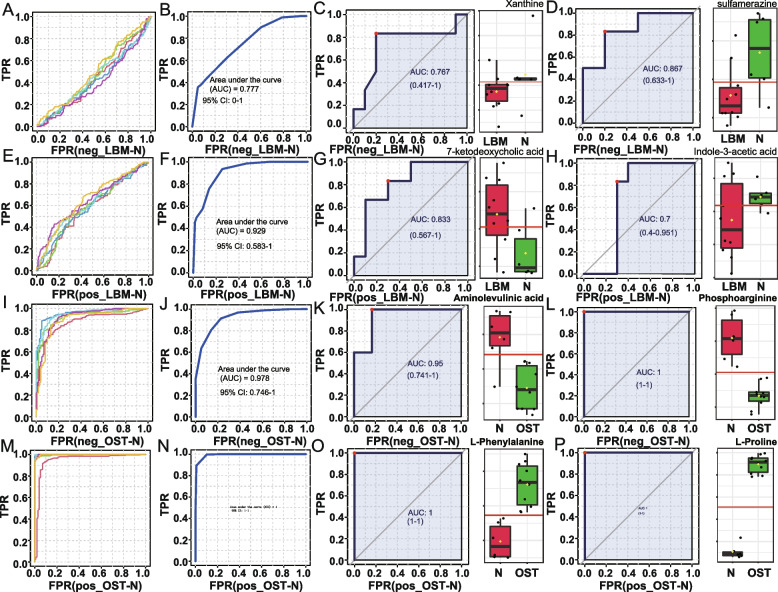
Metabolite markers for pairwise discriminations of OST, LBM and N groups. **A** Receiver operating characteristic (ROC) analysis for the 6 metabolite markers discriminating LBM from N in negetive model. **B** Area under the curve (AUC) applying the 6 LBM vs N metabolite markers to discriminate LBM from N in negetive model. **C** AUC value of metabolite of Xanthine discriminating LBM from N in negetive model. **D** AUC value of metabolite of Sulfamerazine discriminating LBM from N in negetive model. **E** ROC analysis for the 6 metabolite markers discriminating LBM from N in positive model. **F** AUC applying the 6 LBM vs N metabolite markers to discriminate LBM from N in positive model. **G** AUC value of metabolite of 7-ketodeoxycholic acid discriminating LBM from N in positive model. **H** AUC value of metabolite of indole-3-acetic acid discriminating LBM from N in positive model. **I** ROC analysis for the 6 metabolite markers discriminating OST from N in negetive model. **J** AUC applying the 6 OST vs N metabolite markers to discriminate OST from N in negetive model. **K** AUC value of metabolite of Aminolevulinic discriminating OST from N in negetive model. **L** AUC value of metabolite of Phosphoarginine discriminating OST from N in negetive model. **M** ROC analysis for the 6 metabolite markers discriminating OST from N in positive model. **N** AUC applying the 6 OST vs N metabolite markers to discriminate OST from N in positive model. **O**)AUC value of metabolite of L-Phenylalanine discriminating OST from N in positive model. **P** AUC value of metabolite of L-proline discriminating OST from N in positive model. LBM: Lower bone mass, OST: osteoporosis, N: Normal group

We further investigated potential difference metabolites between osteopenia and osteoporosis groups. We built a model for classifying the two groups based on the identified significantly altered metabolites. Our model selected six metabolites to classify and distinguish patients in the LBM from those in the control group in negative ion mode, with an area under the curve (AUC) of 0.777 (Fig. 7A, B) and found that the metabolites xanthine and sulfamerazine were significantly lower in the LBM group than in the control group (*p* < 0.05, Fig. 7C, D). The same six metabolites were identified in the LBM in positive ion mode with an AUC of 0.929 (Fig. 7E, F); however, the metabolites of 7-ketodeoxycholic acid and indole-3-acetic acid in the LBM group were significantly higher than those in the control group (*p* < 0.05) (Fig. 7G, H). To discriminate the OST from the control group, six metabolite markers were identified in the negative ion mode with an AUC of 0.978 (Fig. 7I, J), and aminolevulinic acid and phosphoarginine in were significantly lower in the OST group than in the control group (*p* < 0.05; Fig. 7K, L). Moreover, the distinction of the OST group from the control group in positive ion mode had an AUC of 1.0 (Fig. 7M, N), and the levels of L-phenylalanine and L-proline were higher in the OST group than in the control group (*p* < 0.05; Fig. 7O, P).

### Relationship among the different bacteria, different metabolites, and clinical profiles

**Fig. 8:**
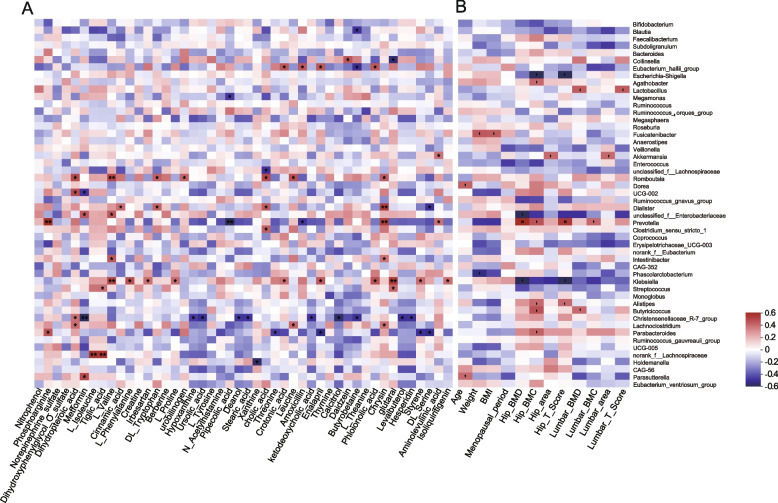
Correlational analyses. **A** Heatmap of the Spearman’s correlation coefficients between gut metabolites and microbiota. **B** Heatmap of the Spearman’s correlation coefficients between clinical parameters and microbiota. *p < 0.05, **p < 0.01, *** *p* < 0.001

We investigated the significant associations among different bacteria, metabolites, and clinical profiles and found that LBM and OST groups enriched with *Klebsiella* were positively correlated with L_Valine, L_Proline, Crotonicacid, Phloionolicacid, Glutara, styrene, isoliquiritigenin, and L_ phenylalanine. *Eubacteriumhallii*group was positively associated with phloionolic and crotonic acids. *Romboutsia* was positively associated with chrysin, leucine, cholic acid, tryptophan, valine, and dihydropteroic acid levels. *Prevotella* was positively correlated with aminolevulinic acid, chrysin, and phosphoarginine levels but negatively associated with amoxicillin and pipecolic acid levels. LBM and OST groups with decreased *Christensenellaceae*_R-7_group were negatively correlated with the levels of hesperidin, levalbutero, butyrobetaine, calcitrio, 7ɑ-ketodeoxycholicacid, stearic acid, deanol, ursolic acid, and hypoxanthine. In addition, we found that the decrease in *Parabacteroides* in the LBM and OST groups was negatively correlated with the levels of threonine, enalapril, styrene, and DL_Serine, but was positively correlated with phosphoarginine (Fig. 8A). In addition, the LBM and OST groups enriched with *Klebsiella* and *Escherichia-Shigella* were negatively correlated with hip BMD, BMC, and T scores. Conversely, LBM and OST groups with a decreased abundance of *Lactobacillus*, *Akkermansia*, *Prevotella*, *Alistipes*, and *Butyricicoccus*, were positively correlated with hip BMD, hip BMC, hip area, hip T-score, lumbar BMD, lumbar BMC, lumbar area, and lumbar T-score (Fig. 8B).

## Discussion

Previous studies have revealed that intestinal flora and their metabolites play an essential in PMO [17]. The intestinal flora affects the development and function of the host immune system by releasing metabolites and immune cells (including T and B cells), stimulating the immune system at the intestinal mucosal barrier, releasing proinflammatory or anti-inflammatory mediators and cytokines, and regulating bone metabolism through blood circulation [18]. In this study, we analyzed and compared the intestinal flora and metabolites of patients with PMO, patients with postmenopausal osteopenia, and healthy individuals. It was obeserved that key intestinal flora and metabolites are disrupted during the development of PMO. Moreover, integrated microbiome and metabolomic analyses showed that interactions between osteoporosis-associated bacteria and metabolites were altered during the progress of PMO. Importantly, we demonstrated the intestinal flora and metabolites for the non-invasive diagnosis of PMO.

The richness and diversity of the gut microbiota are significantly altered in patients with osteoporosis [19]. He et al. have suggested that patients with PMO had lower bacterial richness and diversity than healthy controls [12]. Consistent with this study, our findings suggested decreased bacterial community richness in the PMO group. Moreover, Ling et al. have shown that the gut microbiome of patients with osteoporosis had an increased abundance of *Actinobacillus*, *Blautia*, *Oscillospira*, *Bacteroides*, and *Phascolarctobacterium*, and decreased abundance of *Veillonellaceae*, *Collinsella*, and *Ruminococcaceae* [13]. In our study, we identified a few PMO-related bacterial biomarkers. We observed that the abundance of pathogenic bacteria *Escherichia-Shigella* [20, 21] and *Klebsiella* [22], which are significantly correlated with systemic inflammatory cytokines, was significantly more increased in the PMO than in the control group. Moreover, Ling et al. have suggested that *Blautia* is abundant in patients with osteoporosis group [13], which is consistent with our findings. McGinty et al. reported that *Bifidobacterium* spp. could elevate bone density through increasing the absorption of minerals[23]. However, the abundance of *Bifidobacterium* was enriched in the osteoporosis group in our study. This conflicting result may be attributed to geographical differences. It has been reported that human living in different altitudes and climates may be responsible for the different microbiota compositions [24]. Xianyang, Shaanxi Province, in northwest China, has a continental monsoon climate, with distinct cold, hot, dry, and wet seasons. Meanwhile, as is well known, the diet of Shaanxi people is mainly based on pasta, which may have caused the unique intestinal microbiota of Shaanxi people, thus, this regional factor may also be the reason for obtaining inconsistent results.

In addition, *Lactobacillus* [25]*, Romboutsia* [26], *Akkermansia* [27], *Butyricicoccus* [28], *Ruminococcaceae*_UCG-02, and *Alistipes* [29], which are bacteria involved in the production of short chain fatty acid, were depleted in the LBM and OST groups. Therefore, the enrichment of *Escherichia-Shigella* and *Klebsiella* and the decrease in *Lactobacillus, Romboutsia*, *Akkermansia*, *Butyricicoccus*, *Ruminococcaceae*_UCG-02, and *Alistipes* may predict the development of PMO. Collectively, these results provide insight into the association of intestinal flora with PMO in humans.

The metabolic profile may help identify biomarkers to predict diseases including osteoporosis [13]. In this study, several amino acids, including L-proline, L-threonine, L-serine, L-phenylalanine, and isoleucine, were abundant in the OST group compared to the control group, whereas DL-tryptophan, L-( +)-leucine, and L-tyrosine were depleted in the OST group. Ling et al. have suggested that amino acid metabolism could be a target for intervention in osteoporosis [13], supporting our findings in this study. Interestingly, IAA (downregulated) and 7-KDCA (upregulated) were simultaneously altered in the LBM and OST groups compared with the control group. IAA, has been reported to be produced by the flora from tryptophan metabolism and can alleviate inflammation related to alcoholic liver disease [30] and obesity [31] by directly or indirectly regulating the balance between proinflammatory and anti-inflammatory cytokines (TGF-β, TNF-α, IL-10, and IL-22). Additionally, 7-KDCA is a bile acid of microbial origin, which is associated with advanced stages of fibrosis and non-alcoholic fatty liver disease [32]. Tom et al. [33] reported that patients with chronic liver disease are more likely to develop osteoporosis owing to abnormal vitamin D metabolism, calcium malabsorption, and other factors. Therefore, our results suggest that the simultaneous reduction in IAA and increase in 7-KDCA can directly or indirectly, promote the development of PMO, suggesting that they are potential biomarkers for PMO.

In this study, the pathway enrichment analysis showed that the biosynthesis of aminoacylt-RNA, valine, leucine, and isoleucine, and aromatic amino acid (phenylalanine, tyrosine, and tryptophan), as well as phenylalanine metabolism, were downregulated in the PMO group compared to the healthy control. However, a previous study has suggested that the upregulation of aminoacyl-tRNA biosynthesis pathway is associated with prostate cancer cell development [34]; therefore, this needs to be further studied. Dietary proteins have also been reported to increase calcium absorption [19]. Branched-chain amino acids, including valine, leucine, and isoleucine, were downregulated in the PMO, which is consistent with the study by Ling et al., reporting that patients with osteoporosis had decreased concentrations of serum valine and leucine [13]. Additionally, Isley et al. reported that aromatic amino acid (AAA) intake induces an increase in serum IGF-1 levels, which promotes bone production [19]. In our study, we observed that AAAs, including tyrosine, phenylalanine, and tryptophan, were downregulated in the PMO group. In summary, our results suggest that patients with osteoporosis should consume more proteins, especially food rich in branched-chain and aromatic amino acids.

We further explored the relationships between clinical factors, metabolites, and gut microbiota. Notably, *Klebsiella* and *Escherichia-Shigella* were negatively correlated with hip BMD, hip BMC, and hip T scores. *Klebsiella* has been reported to cause bone and joint infections that are associated with serious morbidity and mortality [19]. *Escherichia-Shigella* was reported to produce propionate ester and other metabolites, thereby inducing the expression of chromatin Acid hydroxylase (Tph) 1 in intestinal chromaffin cells, which increases peripheral 5-HT levels in germ-free mice, promotes osteoclast generation, reduces osteoblast proliferation, and inhibits bone growth [19, 35, 36]. Therefore, targeting *Klebsiella* and *Escherichia-Shigella* in the intestines might delay the progression of osteoporosis. In addition, we found that short-chain fatty acid (SCFA)-producing bacteria, including *Lactobacillus* [37], *Akkermansia* [27], *Prevotella* [38], *Alistipes* [39], and *Butyricicoccus* [28], were positively correlated with hip BMD, hip BMC, hip area, hip T-score, lumbar BMD, lumbar BMC, lumbar area, and lumbar T-score. We also found that *Romboutsia* [26]*,* an SCFA producer, was positively associated with the levels of chrysin, leucine, cholic acid, DL_ tryptophan, L_ valine, and dihydropteroic acid. All above these results suggest a significant interaction between clinical factors, metabolites, and gut microbiota, which might affect the progess of osteoporosis.

This study had some limitations. In this study, all patients were recruited from Xianyang, Shaanxi Province, a small modern city in the mainland area of northwest China. Because the gut microbiota and its metabolites are significantly influenced by geographical, climatic, and dietary habit factors, our findings need validation in other regions. Other limitations are the limited sample size, the cross-sectional design of the study, and the lack of a comprehensive mechanistic analysis and validation cohorts, it is necessary to expand the sample size in the follow-up studies. In the future, the potential identified fecal metabolites and key strains that were associated with PMP need to be validated in vitro or in vivo studies using metagenomic sequencing.

## Conclusion

In summary, this study described the disordered profiles of intestinal bacteria and fecal metabolomes in menopausal patients with osteopenia or osteoporosis. We identified key strains and metabolite differences in the intestinal flora of the participants of this study. We found that the pathogenic bacteria *Klebsiella* and *Escherichia-Shigella* were enriched in patients with LBM and PMO*.* Some SCFAs producers, including *Lactobacillus*, *Akkermansia*, *Prevotella*, *Alistipes*, and *Butyricicoccus*, were reduced in patients with LBM and PMO. Moreover, the metabolites of IAA and 7-KDCA were altered in patients with LBM and PMO. The pathways of aminoacyl-tRNA biosynthesis, valine, leucine, and isoleucine biosynthesis, aromatic amino acid (phenylalanine, tyrosine, and tryptophan) biosynthesis, and phenylalanine metabolism were downregulated in patients with PMO. Additionally, the relationship between these parameters and the bone parameters that can effect osteoporosis is discussed. These findings provide deeper understanding the relationship between gut microbiota, metabolites, and PMO.

## Materials and methods

### Patients and specimen collection

**Fig. 9:**
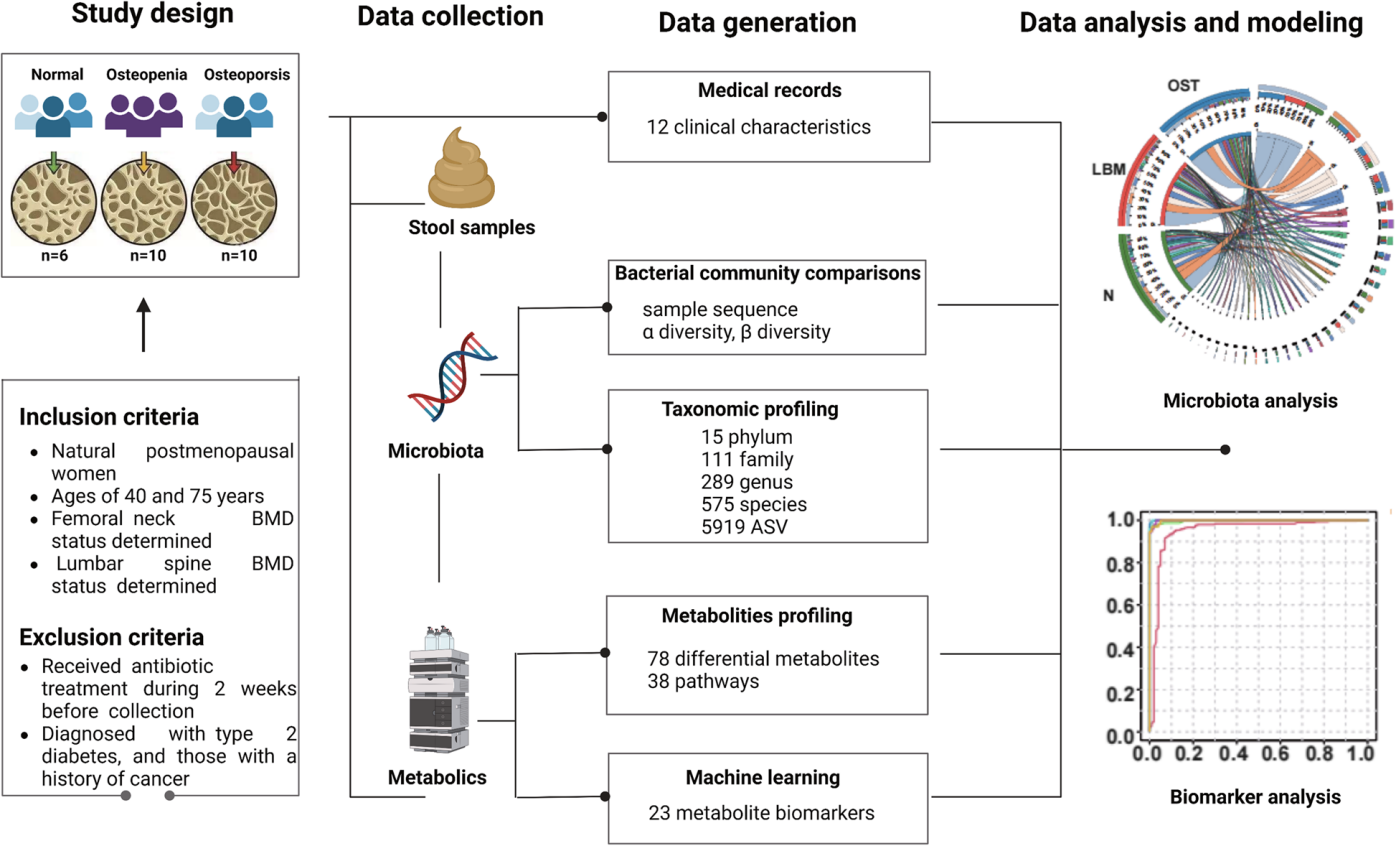
Overview of the prospective study design. A graphical representation summarizing the study design, data collection, and the methodologies of data generation and analysis

All participants were from Shaanxi Province and were admitted to the Affiliated Hospital of Shaanxi University of Traditional Chinese Medicine (Xianyang, China) from October 2021 to December 2021. This trial was registered in the Chinese Clinical Trial Registry (SZFYIEC-PJ-KY-202130). A total of 26 women were recruited for the study based on the inclusion and exclusion criteria (listed below): 10 postmenopausal patients with lower bone mass (LBM: T-score between -1 and -2.5), 10 postmenopausal patients with OST, with T-score less than -2.5, and 6 healthy patients as the control group (Fig. 9). The inclusion criteria and exclusion criteria for the patients were Table 4.

**Table 4:** The inclusion criteria and exclusion criteria for the patients

**Inclusion criteria**
(1) had completed bone mineral density examination and clinical data
(2) naturally postmenopausal women
(3) had no previous systemic diseases
**Exclusion criteria**
(1) secondary osteoporosis
(2) incomplete case data or patients and their families who were unable to complete the questionnaire
(3) under the age of 50 years
(4) use of antibiotics, probiotics, probiotics, or co-organisms within two months before collection of stool samples
(5) patients suffering from severe malnutrition, infections, drug or alcohol abuse
(6) patients with digestive system diseases
(7) patients with complicated with serious systemic diseases, tumors, or other serious primary diseases
(8) patients with previous lumbar and hip surgery history

### Enzyme-linked immunosorbent assay

Sample pretreatment: 50-100 g fecal was collected, and added with the same volume of PBS. The supernatant was centrifuged at 2000 g and stored at -70. The TNF-α, IL-6 and IL-10 were determined by ELISA kits.

### DNA extraction and 16S rRNA gene Sequencing

Total DNA from the fecal samples was extracted using the QIAamp® DNA Stool Mini kit (QIAGEN, Hilden, Germany), according to the manufacturer’s protocol. The DNA quality control and library construction according to the manufacturer’s instructions. Paired-end sequencing was carried out using the Illumina MiSeq (PE300) sequencing platform. The detailed information is described in our previous study [12].

### Sequencing data analysis

After de-multiplexing, the obtained sequences were merged in FLASH (v1.2.11) [40] and quality-filtered using fastp (v0.19.6) [41]. The high-quality sequences were then denoised using the DADA2 [42] plugin in the Qiime2 (v2020.2) pipeline [43]. DADA2–denoised sequences are amplicon sequence variants (ASVs). Finally, sequencing data were analyzed using the free online Majorbio Cloud Platform (www.majorbio.com). 

### Untargeted metabolomics

Fecal metabolites were extracted from fecal samples as described previously, and metabolites were analyzed using UHPLC system (1290; Agilent Technologies, Santa Clara, CA, USA). The detailed information is described in our previous study [12].

### Metabolite data analysis

The mass spectrum data were processed using Compounds Discovered 3.1 software (Thermo Fisher Scientific) for noise reduction, peak alignment, and identification. Differential metabolites were identified using the SIMCA-P v14.1 software (Umetrics AB, Umea, Sweden). PLS-DA analysis method was used to distinguished the metabolites composition of patients with LBM and OST from healthy controls.

### Statistical analysis

Some data are shown as mean ± standard deviation (SD), and some data are shown as mean with quartile. Statistical significance with one-way analysis of variance (ANOVA) followed by Duncan’s multiple comparison test was set at* p* < 0.05. Microbiological analysis was performed using the free online Majorbio Cloud Platform (www.majorbio.com). The metabolite analysis was performed using the free online platform MetaboAnalyst5.0 (www.metaboanalyst.ca/).

## Supplementary Information

**Additional file 1:** **Supplemental Table 1.** Result from pathwayanalysis between LBM & N. **SupplementalTable 2.** Result frompathway analysis between OST & N. **Fig. S1.** Changes in the composition ofkey metabolites among OST, LBM and N groups. LBM: Lower bone mass, OST:osteoporosis, N: Normal group.

## Data Availability

The detail data and materials available please see https://www.ncbi.nlm.nih.gov/sra/PRJNA916764. The amplicon sequencing data are available in National Center for Biotechnology Information with accession numbers of Sequence Read Archive (SRA) submission: PRJNA916764.

## Abbreviations

PMO: Postmenopausal osteoporosis
LBM: Lower bone mass
OST: Postmenopausal women with osteoporosis
SCFAs: Short-chain fatty acid
AUC: Area under the curve
IL-10: Interleukin-10
TNF-α: Tumor necrosis factor-α
IL-6: Interleukin-6
BMD: Bone mineral density
BMC: Bone marrow concentrate
NMDS: Nonmetric multidimensional scaling
LC–MS: Liquid chromatography-mass spectrometry
7-KDCA: 7-Ketodeoxycholic acid
IAA: Indole-3-acetic acid
AAA: Aromatic amino acid
SD: Standard deviation
ANOVA: One-way analysis of variance
SRA: Sequence Read Archive
