# Supplementary material for: The potential of Klebsiella and Escherichia-Shigella and amino acids metabolism to monitor patients with postmenopausal osteoporosis in northwest China
Source: BMC Microbiol. 2023 Jul 26;23:199. doi: 10.1186/s12866-023-02927-5 (PMC10373412; doi:10.1186/s12866-023-02927-5)
Supplement: Supplementary file 1 — Additional file 1: Supplemental Table 1. Result from pathwayanalysis between LBM & N. SupplementalTable 2. Result frompathway analysis between OST & N. Fig. S1. Changes in the composition ofkey metabolites among OST, LBM and N groups. LBM: Lower bone mass, OST:osteoporosis, N: Normal group. [file 12866_2023_2927_MOESM1_ESM.docx]

The potential of *Klebsiella* and *Escherichia-Shigella* and amino acids metabolism to monitor patients with postmenopausal osteoporosis in northwest China

Zhuang Liang^1,#^, Yuqi Hao^3,#^, Lei Yang^1,#^, Puwei Yuan^1^, Wulin Kang^1^, Tingting Liang^2,#^, Bing Gu^2*^, and Bo Dong^1,*^

^1^Department of Rehabilitation Hospital Pain Ward, Xi'an Jiaotong University Affiliated Honghui Hospital, Xi'an, Shaanxi, 710054, China

^2^Department of Clinical Laboratory Medicine, Guangdong Provincial People’s Hospital (Guangdong Academy of Medical Sciences), Southern Medical University, Guangzhou, Guangdong, 510000, China

^3^Department of Internal Medicine, Ordos Traditional Chinese Medicine Hospital, Ordos, Inner Mongolia, 017000, China

***Correspondence:** Tingting Liang, Bing Gu, Bo Dong

Corresponding Author: 1320199507@qq.com; dahe_78@163.com; [gubing@gdph.org.cn](mailto:gubing@gdph.org.cn;)

**Supplemental Table 1.** Result from pathway analysis between LBM & N.

|  | Total Cmpd | Hits | Raw p | -log10(p) | Holm adjust | FDR | Impact |
| --- | --- | --- | --- | --- | --- | --- | --- |
| Folate biosynthesis | 27 | 1 | 3.78E-02 | 1.42E+00 | 2.27E-01 | 2.27E-01 | 0.00 |
| Purine metabolism | 65 | 1 | 1.92E-01 | 7.17E-01 | 9.59E-01 | 5.65E-01 | 0.01 |
| Porphyrin and chlorophyll metabolism | 30 | 1 | 3.84E-01 | 4.16E-01 | 1.00E+00 | 5.65E-01 | 0.03 |
| Glycine, serine and threonine metabolism | 33 | 1 | 3.84E-01 | 4.16E-01 | 1.00E+00 | 5.65E-01 | 0.00 |
| Tyrosine metabolism | 42 | 1 | 5.37E-01 | 2.70E-01 | 1.00E+00 | 5.65E-01 | 0.01 |
| Sulfur metabolism | 8 | 1 | 5.65E-01 | 2.48E-01 | 1.00E+00 | 5.65E-01 | 0.32 |
| Arginine and proline metabolism | 38 | 7 | 6.93E-02 | 1.16E+00 | 1.00E+00 | 5.22E-01 | 0.37 |
| Glutathione metabolism | 28 | 3 | 7.51E-02 | 1.12E+00 | 1.00E+00 | 5.22E-01 | 0.03 |
| Porphyrin and chlorophyll metabolism | 30 | 2 | 7.87E-02 | 1.10E+00 | 1.00E+00 | 5.22E-01 | 0.03 |
| Arginine biosynthesis | 14 | 4 | 1.30E-01 | 8.84E-01 | 1.00E+00 | 5.22E-01 | 0.41 |
| Ubiquinone and other terpenoid-quinone biosynthesis | 9 | 1 | 1.49E-01 | 8.27E-01 | 1.00E+00 | 5.22E-01 | 0.00 |
| Alanine, aspartate and glutamate metabolism | 28 | 2 | 1.51E-01 | 8.21E-01 | 1.00E+00 | 5.22E-01 | 0.28 |
| Butanoate metabolism | 15 | 2 | 1.51E-01 | 8.21E-01 | 1.00E+00 | 5.22E-01 | 0.03 |
| Cysteine and methionine metabolism | 33 | 1 | 1.97E-01 | 7.05E-01 | 1.00E+00 | 5.22E-01 | 0.10 |
| Biosynthesis of unsaturated fatty acids | 36 | 4 | 2.01E-01 | 6.97E-01 | 1.00E+00 | 5.22E-01 | 0.00 |
| Tryptophan metabolism | 41 | 2 | 2.49E-01 | 6.04E-01 | 1.00E+00 | 5.22E-01 | 0.01 |
| Glycerophospholipid metabolism | 36 | 2 | 2.51E-01 | 6.01E-01 | 1.00E+00 | 5.22E-01 | 0.03 |
| Steroid biosynthesis | 42 | 3 | 2.56E-01 | 5.92E-01 | 1.00E+00 | 5.22E-01 | 0.07 |
| Pantothenate and CoA biosynthesis | 19 | 3 | 2.58E-01 | 5.89E-01 | 1.00E+00 | 5.22E-01 | 0.01 |
| D-Glutamine and D-glutamate metabolism | 6 | 1 | 2.60E-01 | 5.85E-01 | 1.00E+00 | 5.22E-01 | 0.50 |
| Glyoxylate and dicarboxylate metabolism | 32 | 1 | 2.60E-01 | 5.85E-01 | 1.00E+00 | 5.22E-01 | 0.00 |
| Nitrogen metabolism | 6 | 1 | 2.60E-01 | 5.85E-01 | 1.00E+00 | 5.22E-01 | 0.00 |
| Phenylalanine, tyrosine and tryptophan | 4 | 2 | 2.66E-01 | 5.75E-01 | 1.00E+00 | 5.22E-01 | 1.00 |
| biosynthesis |  |  |  |  |  |  |  |
| Phenylalanine metabolism | 10 | 2 | 2.66E-01 | 5.75E-01 | 1.00E+00 | 5.22E-01 | 0.36 |
| Purine metabolism | 65 | 7 | 2.67E-01 | 5.74E-01 | 1.00E+00 | 5.22E-01 | 0.06 |
| Glycine, serine and threonine metabolism | 33 | 5 | 2.75E-01 | 5.61E-01 | 1.00E+00 | 5.22E-01 | 0.00 |
| Tyrosine metabolism | 42 | 2 | 3.13E-01 | 5.04E-01 | 1.00E+00 | 5.67E-01 | 0.16 |
| Nicotinate and nicotinamide metabolism | 15 | 2 | 4.28E-01 | 3.69E-01 | 1.00E+00 | 7.39E-01 | 0.19 |
| Aminoacyl-tRNA biosynthesis | 48 | 9 | 4.49E-01 | 3.47E-01 | 1.00E+00 | 7.42E-01 | 0.00 |
| Thiamine metabolism | 7 | 1 | 4.79E-01 | 3.20E-01 | 1.00E+00 | 7.58E-01 | 0.00 |
| Histidine metabolism | 16 | 4 | 5.31E-01 | 2.75E-01 | 1.00E+00 | 8.07E-01 | 0.31 |
| Valine, leucine and isoleucine degradation | 40 | 3 | 6.17E-01 | 2.09E-01 | 1.00E+00 | 8.80E-01 | 0.00 |
| Valine, leucine and isoleucine biosynthesis | 8 | 4 | 7.00E-01 | 1.55E-01 | 1.00E+00 | 8.80E-01 | 0.00 |
| Taurine and hypotaurine metabolism | 8 | 1 | 7.02E-01 | 1.54E-01 | 1.00E+00 | 8.80E-01 | 0.00 |
| Primary bile acid biosynthesis | 46 | 4 | 7.11E-01 | 1.48E-01 | 1.00E+00 | 8.80E-01 | 0.03 |
| Pyrimidine metabolism | 39 | 2 | 7.63E-01 | 1.17E-01 | 1.00E+00 | 8.80E-01 | 0.11 |
| Starch and sucrose metabolism | 18 | 1 | 7.69E-01 | 1.14E-01 | 1.00E+00 | 8.80E-01 | 0.07 |
| Fatty acid degradation | 39 | 2 | 7.87E-01 | 1.04E-01 | 1.00E+00 | 8.80E-01 | 0.00 |
| beta-Alanine metabolism | 21 | 2 | 7.89E-01 | 1.03E-01 | 1.00E+00 | 8.80E-01 | 0.00 |
| Glycerolipid metabolism | 16 | 1 | 8.11E-01 | 9.11E-02 | 1.00E+00 | 8.80E-01 | 0.24 |
| Galactose metabolism | 27 | 1 | 8.11E-01 | 9.11E-02 | 1.00E+00 | 8.80E-01 | 0.00 |
| Lysine degradation | 25 | 4 | 9.21E-01 | 3.55E-02 | 1.00E+00 | 9.73E-01 | 0.00 |
| Fatty acid biosynthesis | 47 | 1 | 9.96E-01 | 1.78E-03 | 1.00E+00 | 9.96E-01 | 0.01 |
| Fatty acid elongation | 39 | 1 | 9.96E-01 | 1.78E-03 | 1.00E+00 | 9.96E-01 | 0.00 |

**Supplemental Table 2**. Result from pathway analysis between OST & N.

|  | Total Cmpd | Hits | Raw p | -log10(p) | Holm adjust | FDR | Impact |
| --- | --- | --- | --- | --- | --- | --- | --- |
| Purine metabolism | 65 | 1 | 1.10E-04 | 3.96E+00 | 6.58E-04 | 6.58E-04 | 0.01 |
| Porphyrin and chlorophyll metabolism | 30 | 1 | 1.19E-03 | 2.92E+00 | 5.97E-03 | 2.39E-03 | 0.03 |
| Glycine, serine and threonine metabolism | 33 | 1 | 1.19E-03 | 2.92E+00 | 5.97E-03 | 2.39E-03 | 0.00 |
| Folate biosynthesis | 27 | 1 | 5.13E-03 | 2.29E+00 | 1.54E-02 | 7.69E-03 | 0.00 |
| Sulfur metabolism | 8 | 1 | 1.41E-01 | 8.51E-01 | 2.82E-01 | 1.69E-01 | 0.32 |
| Tyrosine metabolism | 42 | 1 | 3.99E-01 | 3.99E-01 | 3.99E-01 | 3.99E-01 | 0.01 |
| Aminoacyl-tRNA biosynthesis | 48 | 9 | 2.56E-10 | 9.59E+00 | 9.72E-09 | 9.72E-09 | 0.00 |
| Valine, leucine and isoleucine biosynthesis | 8 | 4 | 2.40E-08 | 7.62E+00 | 8.87E-07 | 2.88E-07 | 0.00 |
| Phenylalanine, tyrosine and tryptophan biosynthesis | 4 | 2 | 3.03E-08 | 7.52E+00 | 1.09E-06 | 2.88E-07 | 1.00 |
| Phenylalanine metabolism | 10 | 2 | 3.03E-08 | 7.52E+00 | 1.09E-06 | 2.88E-07 | 0.36 |
| Lysine degradation | 25 | 4 | 6.55E-08 | 7.18E+00 | 2.23E-06 | 4.98E-07 | 0.00 |
| Glycine, serine and threonine metabolism | 33 | 5 | 1.20E-07 | 6.92E+00 | 3.97E-06 | 7.61E-07 | 0.00 |
| Primary bile acid biosynthesis | 46 | 4 | 1.02E-05 | 4.99E+00 | 3.28E-04 | 5.56E-05 | 0.03 |
| Valine, leucine and isoleucine degradation | 40 | 3 | 2.28E-05 | 4.64E+00 | 7.07E-04 | 1.08E-04 | 0.00 |
| Pyrimidine metabolism | 39 | 2 | 7.11E-05 | 4.15E+00 | 2.13E-03 | 3.00E-04 | 0.11 |
| Arginine and proline metabolism | 38 | 7 | 8.11E-05 | 4.09E+00 | 2.35E-03 | 3.08E-04 | 0.37 |
| Porphyrin and chlorophyll metabolism | 30 | 2 | 1.52E-04 | 3.82E+00 | 4.26E-03 | 5.26E-04 | 0.03 |
| Tyrosine metabolism | 42 | 2 | 1.85E-04 | 3.73E+00 | 5.00E-03 | 5.87E-04 | 0.16 |
| Glycerophospholipid metabolism | 36 | 2 | 2.36E-04 | 3.63E+00 | 6.13E-03 | 6.42E-04 | 0.03 |
| Ubiquinone and other terpenoid-quinone biosynthesis | 9 | 1 | 2.37E-04 | 3.63E+00 | 6.13E-03 | 6.42E-04 | 0.00 |
| Nicotinate and nicotinamide metabolism | 15 | 2 | 2.36E-03 | 2.63E+00 | 5.65E-02 | 5.97E-03 | 0.19 |
| Biosynthesis of unsaturated fatty acids | 36 | 4 | 5.18E-03 | 2.29E+00 | 1.19E-01 | 1.23E-02 | 0.00 |
| Fatty acid degradation | 39 | 2 | 9.65E-03 | 2.02E+00 | 2.12E-01 | 2.16E-02 | 0.00 |
| Tryptophan metabolism | 41 | 2 | 1.53E-02 | 1.82E+00 | 3.21E-01 | 3.22E-02 | 0.01 |
| Starch and sucrose metabolism | 18 | 1 | 1.65E-02 | 1.78E+00 | 3.29E-01 | 3.29E-02 | 0.07 |
| Purine metabolism | 65 | 7 | 2.16E-02 | 1.67E+00 | 4.11E-01 | 3.92E-02 | 0.06 |
| beta-Alanine metabolism | 21 | 2 | 2.22E-02 | 1.65E+00 | 4.11E-01 | 3.92E-02 | 0.00 |
| Alanine, aspartate and glutamate metabolism | 28 | 2 | 2.37E-02 | 1.62E+00 | 4.11E-01 | 3.92E-02 | 0.28 |
| Butanoate metabolism | 15 | 2 | 2.37E-02 | 1.62E+00 | 4.11E-01 | 3.92E-02 | 0.03 |
| Taurine and hypotaurine metabolism | 8 | 1 | 3.35E-02 | 1.48E+00 | 5.02E-01 | 4.99E-02 | 0.00 |
| Arginine biosynthesis | 14 | 4 | 3.38E-02 | 1.47E+00 | 5.02E-01 | 4.99E-02 | 0.41 |
| Steroid biosynthesis | 42 | 3 | 3.42E-02 | 1.47E+00 | 5.02E-01 | 4.99E-02 | 0.07 |
| Fatty acid biosynthesis | 47 | 1 | 4.58E-02 | 1.34E+00 | 5.50E-01 | 6.22E-02 | 0.01 |
| Fatty acid elongation | 39 | 1 | 4.58E-02 | 1.34E+00 | 5.50E-01 | 6.22E-02 | 0.00 |
| Pantothenate and CoA biosynthesis | 19 | 3 | 9.01E-02 | 1.05E+00 | 9.01E-01 | 1.18E-01 | 0.01 |
| Histidine metabolism | 16 | 4 | 1.58E-01 | 8.00E-01 | 1.00E+00 | 2.01E-01 | 0.31 |
| Glutathione metabolism | 28 | 3 | 1.69E-01 | 7.71E-01 | 1.00E+00 | 2.08E-01 | 0.03 |
| Glycerolipid metabolism | 16 | 1 | 2.27E-01 | 6.44E-01 | 1.00E+00 | 2.61E-01 | 0.24 |
| Galactose metabolism | 27 | 1 | 2.27E-01 | 6.44E-01 | 1.00E+00 | 2.61E-01 | 0.00 |
| Cysteine and methionine metabolism | 33 | 1 | 3.35E-01 | 4.75E-01 | 1.00E+00 | 3.74E-01 | 0.10 |
| Thiamine metabolism | 7 | 1 | 5.79E-01 | 2.37E-01 | 1.00E+00 | 6.29E-01 | 0.00 |
| D-Glutamine and D-glutamate metabolism | 6 | 1 | 9.79E-01 | 9.09E-03 | 1.00E+00 | 9.79E-01 | 0.50 |
| Glyoxylate and dicarboxylate metabolism | 32 | 1 | 9.79E-01 | 9.09E-03 | 1.00E+00 | 9.79E-01 | 0.00 |
| Nitrogen metabolism | 6 | 1 | 9.79E-01 | 9.09E-03 | 1.00E+00 | 9.79E-01 | 0.00 |


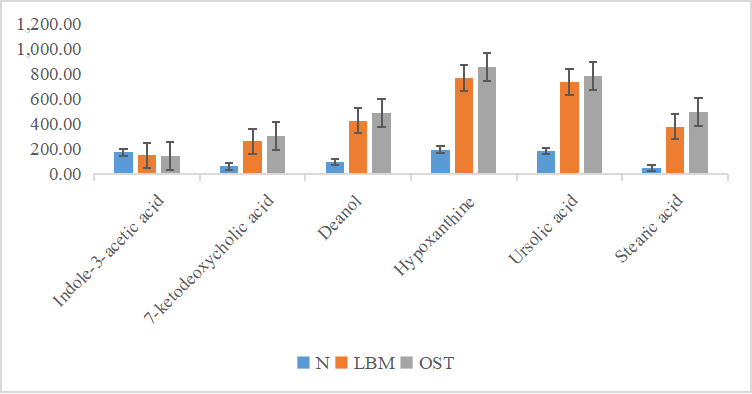


**Fig. S1.** Changes in the composition of key metabolites among OST, LBM and N groups. LBM: Lower bone mass, OST: osteoporosis, N: Normal group.
